# Pyruvate Kinase 2, an Energy Metabolism Related Enzyme, May Have a Neuroprotective Function in Retinal Degeneration

**DOI:** 10.1177/17590914231151534

**Published:** 2023-02-17

**Authors:** Jiaming Zhou, Per Ekström

**Affiliations:** 1 Ophthalmology, Department of Clinical Sciences, 5193 Lund University, Lund, Sweden

**Keywords:** PKM2, retinal degeneration, cell death, TUNEL, organotypic retinal explant culture

## Abstract

Retinitis pigmentosa (RP) is an inherited disorder that results in vision impairment but general and mutation-independent therapeutic strategies are not available. However, it is widely regarded that the cGMP system, including cGMP and its interactor cGMP-dependent protein kinase (PKG), acts as a crucial effector during retinal degeneration. We have previously identified a list of cGMP-PKG-dependent genes in the context of RP, and in this study, we further validated one of these, namely pyruvate kinase 2 (PKM2), and investigated the potential role of PKM2 for the photoreceptors’ well-being during RP. With the aid of organotypic retinal explant cultures, we pharmacologically manipulated the PKM2 activities in two different RP mouse models (*rd2* and *rd10*) via the addition of TEPP-46 (a PKM2 activator) and found that activation of PKM2 alleviates the progress of photoreceptor death in the *rd10* mouse model. We also noted that the expression of both PKM2 and one of its targets, glucose transporter-1 (Glut1), showed alterations depending on the degeneration state. The observations provide supportive evidence that PKM2 may serve as a novel potential molecular target in RP.

## Introduction

Retinitis pigmentosa (RP) refers to a group of genetic disorders in retinal photoreceptors and is one of the most common forms of inherited retinal degeneration, leading to severe vision problems. The prevalence of RP reaches 1 in 3000–4000 (Haim, 2002), and there is currently in principle no effective treatment available, likely because of the extensively heterogeneous mutations of disease-leading genes (Daiger et al., 2013). Increased photoreceptor levels of the signaling molecule cGMP have been reported in several mouse models for RP, including *rd2* and *rd10* (Arango-Gonzalez et al., 2014; Power et al., 2020). Moreover, work in our laboratory has previously suggested a neurodegenerative effect of such elevated cGMP, that at least in part works via increasing the activity of its dependent protein kinase G (cGMP-dependent protein kinase; PKG), leading to over-phosphorylation within photoreceptors (Paquet-Durand et al., 2009).

The M2 subtype of pyruvate kinase (PKM2) has been shown to be expressed within photoreceptors (Rajala et al., 2018) and our previous transcriptome research (Zhou et al., 2021), where we modulated the cGMP-PKG system in retinal explants, showed a lower expression of the PKM2 gene after PKG activation in wild-type (WT) retinas. This pointed to the possibility that PKM2 may be repressed by the high cGMP-PKG activity within RP photoreceptors. We know that the same type of PKG activation leads to photoreceptor degeneration in otherwise healthy WT retinas (Paquet-Durand et al., 2009), and that PKG inhibition via a liposome-formulated cGMP-analogue can protect degenerating photoreceptors and help preserve the retinal function in the *rd2* and *rd10* models after systemic administration (Vighi et al., 2018). We therefore speculated that since PKG may act to decrease PKM2 in the degenerating photoreceptors, a direct activation of PKM2 during the degeneration may have neuroprotective effects.

To acquire a better understanding of this, we here utilized a manipulation of the PKM2 activity in the retina via a pharmacological method. To do so we added 6-((3-Aminophenyl)methyl)-4-methyl-2-methylsulfinyl­thieno[3,4]-pyrrolo[1,3-d]pyridazin-5-one (TEPP-46, a PKM2 activator) (Jiang et al., 2010) during organotypic retinal explant culturing with tissue from either the *rd2* or the *rd10* mouse strains (Chang et al., 2002), two RP models where photoreceptor cGMP is high (Arango-Gonzalez et al., 2014). According to our hypothesis, we expected to have a lower expression of PKM2 in these disease models, and that activation of this enzyme, which as one of its target has the energy metabolism related glucose transporter-1 (Glut1; Luo & Semenza, 2012), may alleviate the progress of cell death.

Our investigations show that *rd10* retinas benefitted with an apparent lower amount of photoreceptor death from PKM2 activation, while no difference could be observed in the *rd2* strain with the same treatment. Given the slow progress of photoreceptor loss in the *rd2* strain, a positive effect of long-term PKM2 activation in *rd2* cannot be excluded, though.

## Materials and Methods

### Animals

The mouse strains *rd2* (Sanyal et al., 1980), *rd10* (C57BL/6, RRID:MGI:3581193, The Jackson Laboratory), and wild-type (WT; C3H, Sanyal & Bal, 1973), all from own colonies, were used in our study. Animals were kept under standard white cyclic lighting, with ad libitum access to food and water, and used irrespective of sex. All procedures had been evaluated by the local animal care and ethics committees and performed in accordance with permit 2124-20. All efforts were made to keep the number of animals used and their suffering to a minimum. Day of birth of the animal was considered as postnatal 0 (P0), with the day following this considered as P1, etc.

### Organotypic Retinal Explant Culture

Retinas from *rd2* and *rd10* mice at the age of P9 were used to generate explants following the standard protocol in our lab (Zhou et al., 2021). In brief, inserts with the explants were put into six-well culture plates with 1.5 mL serum-free medium in each well. Plates were incubated at 37°C with a 5% CO_2_ atmosphere, and the medium was replaced every two days. No additions were made to the cultures in the first 2 days. After this, i.e., at a time that equals P11, the cultures were treated with 50 µM PKM2 activator (TEPP-46 (HY-18657), MedChemExpress, Monmouth Junction, New Jersey, USA) for 8 days, with medium changes every second day during the whole culturing period. Hence, the culturing paradigm used was termed P9 + 2 + 8. In parallel, untreated counterparts served as controls receiving the same amount of solvent (H_2_O). The culturing paradigm thus means that the endpoint resembles P19.

### Cryosectioning

Retinal tissues from *rd2, rd10* and WT in *vivo* at P9 and P19, as well as the cultured explants from *rd2* and *rd10* at the culture endpoint P19, were collected for cryosectioning. All samples were treated with 4% formaldehyde for 2 h, washed 3 *×* 15* m*in in phosphate-buffered saline (PBS), cryoprotected in PBS + 10% sucrose overnight at 4°C, and then with PBS + 25% sucrose for 2 h. After embedding in a medium with 30% bovine serum albumin (BSA; Cat. No.: A5253-250G; Sigma-Aldrich, St. Louis, MO, USA) and 3% gelatine (Cat. No.: 1040781000, Merck Millipore, Burlington, MA, USA) mixed in H_2_O, 12-μm-thick retinal cross-sections were cut and collected from a HM560 cryotome (Microm, Walldorf, Germany). The sections were stored at −20°C for later usage. Cryosections were then used for immunostaining and TUNEL assay.

### Immunostaining

For immunostaining, briefly, the cryosections were dried at room temperature for 15 min and rehydrated in PBS. They were then blocked with 1% BSA + 0.25% Triton × 100 + 5% goat serum in PBS at room temperature for 45* m*in. The primary antibodies anti-PKM2 (1:200, Cat No.: 4053, RRID: AB_1904096, Cell Signaling Technology, Danvers, Massachusetts, USA), anti-GLUT1 (1:200, Cat No.: MABS132, RRID: AB_2571629, Sigma-Aldrich, St. Louis, MO, USA) and anti-Phospho-CREB (1:200, Cat No.: MA5-11192, RRID: AB_10986840, ThermoFisher, Scientific, Waltham, Massachusetts, USA) were diluted with 1% BSA and 0.25% Triton × 100 in PBS (PTX) and incubated at 4°C for overnight; a no primary antibody control ran in parallel. Sections were washed 3 × 5* m*in, each in PTX and incubated with a goat anti-rabbit IgG (H + L) highly cross-adsorbed secondary antibody, Alexa Fluor™ Plus 488 (1:800, Cat No.: A32731, RRID: AB_2633280, Thermo Fisher Scientific, Waltham, MA, USA) and Chicken anti-Mouse IgG (H + L) Cross-Adsorbed Secondary Antibody, Alexa Fluor™ 594 (1:200, Cat No.: A-21201, RRID: AB_2535787, Thermo Fisher Scientific, Waltham, MA, USA), goat anti-rabbit IgG (H + L) cross-adsorbed secondary antibody with Alexa Fluor 594 (1:800, Cat No.: A11037, RRID: AB_2534095, Thermo Fisher Scientific, Waltham, MA, USA) at 1:800 dilution in PTX. After 3 *×* 5-min. PBS washes, the sections were mounted with Vectashield DAPI (Vector, Burlingame, CA, USA).

### TUNEL Assay

Fluorescent terminal deoxynucleotidyl transferase dUTP nick end labeling (TUNEL) assay (11687495910, Roche Diagnostics, Mannheim, Germany) was performed as by manufacturer's instructions and used on cryosections from the different conditions to evaluate cell death. The number of TUNEL-positive cells in the photoreceptor bearing outer nuclear layer (ONL) was calculated as shown previously (Paquet-Durand et al., 2009).

### Microscopy and Image Processing

A Zeiss Imager Z1 Apotome Microscope (Zeiss, Oberkichen, Germany), with a Zeiss Axiocam digital camera was used for microscopy observations. Image generation and contrast enhancement were performed identically for all images via the ZEN2 software (blue edition). The immunostaining was analyzed for staining differences via three sections each from three to five animals for each condition, after which the fluorescent intensities of positive cells randomly distributed within in the area of interest (the ONL, i.e., the photoreceptor layer, plus the area represented by the photoreceptor segments) were assessed. Fluorescence intensity was captured and analyzed by the ImageJ software (version 1.53a, NIH, MD, USA). The freehand selection function was used to target the ONL or segments, after which the fluorescence intensity was calculated with the measure function.

For both the immunostaining and TUNEL analyses, three to four sections from a given retina gave rise to an average, which was then combined with the corresponding averages from the same group (in most cases four to five animals) to give the final average for the various conditions.

### Statistics

All ANOVA tests and one-tailed t-tests in this study were performed via the R software (downloaded online from https://www.r-project.org/) and a p-value lower than 0.05 was regarded as significant. Specifically, the function “compare_means” was used, with the argument of “methods” being set as either “anova” or “t.test”. In the t-test, the argument of “alternative” of the same function of “compare_means” was set as either “greater” or “less” according to the specific hypothesis.

## Results

**Figure 1.**
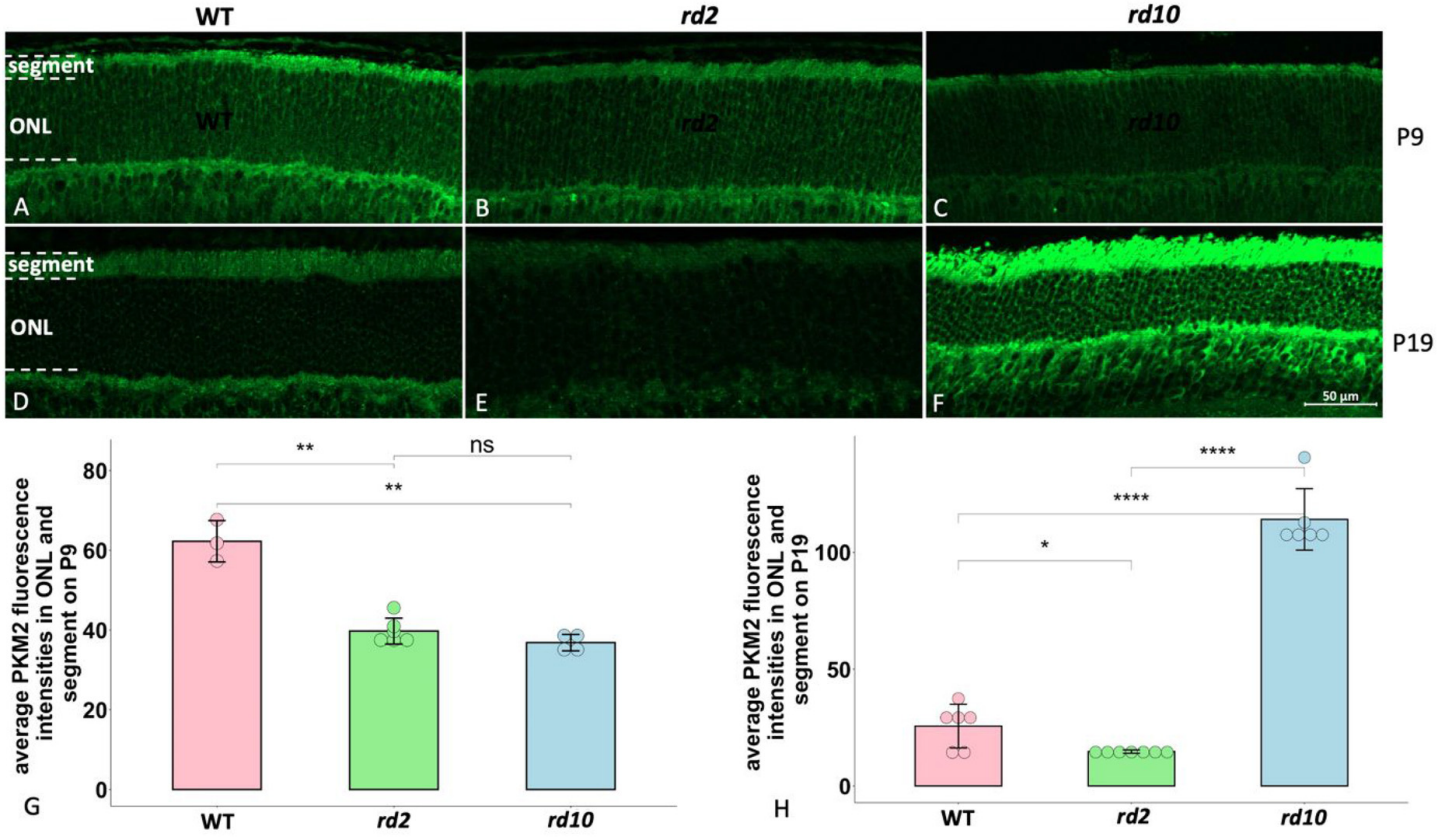
Comparisons of PKM2 expression among WT, *rd2*, and *rd10* on P9 and P19. PKM2 fluorescence staining is in green. A-C: PKM2 expression in ONL and segments from WT, *rd2* and *rd10*, respectively, at P9; D-F: PKM2 expression in ONL and segments from WT, *rd2*, and *rd10* at P19. G-H: Bar chart summarizing the PKM2 fluorescence intensities among the three different strains on P9 and P19, respectively. The ANOVA with Dunnett's post hoc analysis was used, n = 3–6 biological replicates. *p < 0.05, **p < 0.01, ****p < 0.0001. ONL = outer nuclear layer.

In the present study, we investigated how PKM2 appears during retinal degeneration, and compared the PKM2 expression within photoreceptors in the two RP models and also in wild-type (WT) on P9 and P19, which are equivalent to the start and end points, respectively, of our culturing protocol (all data obtained from the explant cultures are presented below). We found that PKM2 is mainly expressed in the segment area, where the inner and outer segments of the photoreceptors are situated. On P9, we observed a lower PKM2 expression of photoreceptors in *rd2* as well as *rd10* than in WT (Figure 1A–C, G). On P19 a similar pattern of lower PKM2 expression was observed in *rd2* compared to WT, while, by contrast, it was apparently higher expressed in *rd10* than in its WT counterpart (Figure 1D–F, H). To evaluate the degeneration of the different strains, we compared the ONL thickness of each strain between P9 and P19 (Table 1). We found a slight and clear decrease of ONL thickness in *rd2* and *rd10* between these two time points, respectively, while we could not observe any ONL difference in WT.

**Table 1.** Comparisons of ONL Thickness between the Same Strains at P9 and P19.

	WT	*rd2*	*rd10*
P9	67 ± 4	66 ± 1	74 ± 5
P19	65 ± 6	61 ± 4	28 ± 5
p value	0.571	0.023	0.000

Data are presented as Mean ± SD, and units are µm.

ONL = outer nuclear layer.

**Figure 2.**
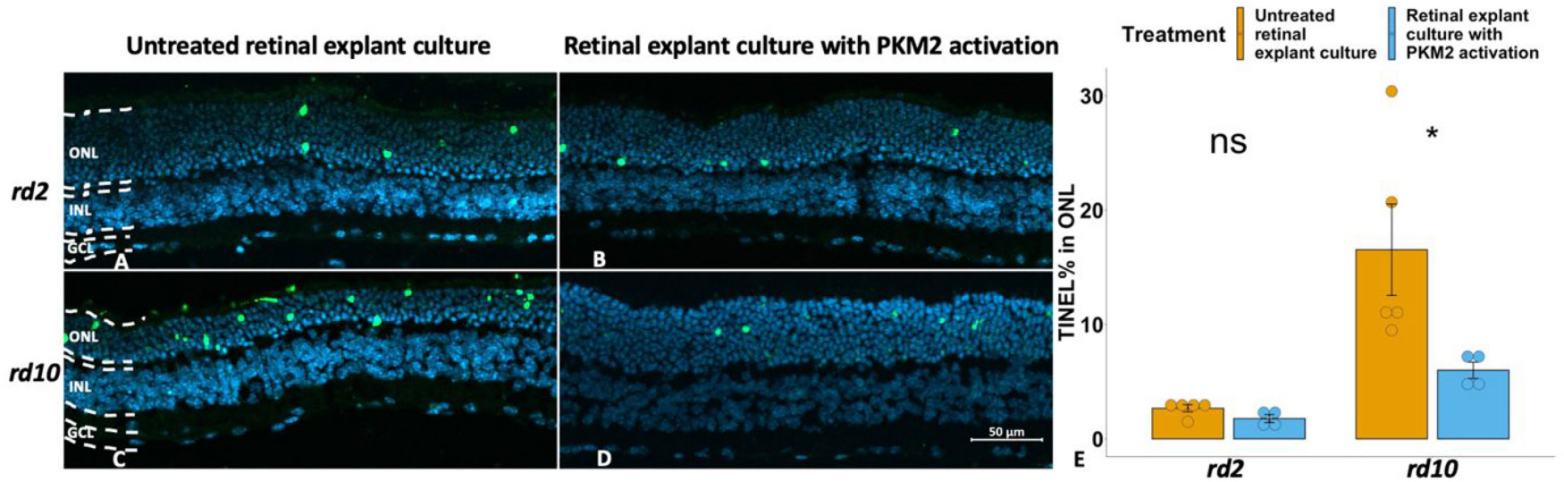
Activation of PKM2 showed a decreased number of dying photoreceptors in *rd10* explants, while the same treatment did not change these parameters in *rd2* explants. TUNEL-positive cells are shown in green, and DAPI (blue) was used as nuclear counterstain. A: *rd2* untreated control; B: *rd2* treated with TEPP-46 (PKM2 activator); C: *rd10* untreated control; D: *rd10* treated with TEPP-46; E: Bar chart summarizing the TUNEL-positive ONL cell counts of the different experimental groups, respectively. ONL = outer nuclear layer; INL = inner nuclear layer; GCL = ganglion cell layer. The t-test was used as described in Materials and methods section, n = 4–5 biological replicates. *p < 0.05.

To explore whether PKM2 has any neuroprotective effects during retinal degeneration, we manipulated the PKM2 activities within the photoreceptors in the two RP models by adding TEPP-46 during the organotypic retinal explant culturing. For the *rd10* model, we found that the number of TUNEL-positive cells decreased compared to the untreated counterpart (Figure 2C–E). By contrast, we could not observe any significant differences in photoreceptor death in the explants with PKM2 activation from the *rd2* model compared to their untreated counterparts (Figure 2A–B, E), although a reduction that came close to being statistically significant was observed. For clarity, the individual values for this (TUNEL) and the other two studied parameters below in the explant treatment studies (PKM2 and Glut1 expression, respectively) are given in Table 2.

**Table 2.** Compilation of All Values from the Retinal Explant Experiments, Arranged so That the Outcome for the Various Parameters (TUNEL, PKM2, Glut1) Can Be Compared Between the Individual Samples. * = p < 0.05, ** = p < 0.01.

*rd2* untreated	TUNEL	PKM2	Glut1
Sample 1	2,9	11,2	31,9
Sample 2	3,3	13,0	35,0
Sample 3	3,1	14,5	36,7
Sample 4	2,6	15,9	47,4
Sample 5	1,5	19,2	52,5
Mean ± SD	2,7 ± 0,6	14,8 ± 2,7	40,7 ± 7,9
*rd2* + TEPP-46	TUNEL	PKM2	Glut1
Sample 1	1,6	26,2	52,1
Sample 2	2,6	31,7	57,4
Sample 3	2,0	32,2	67,0
Sample 4	0,9		71,0
Mean ± SD	1,8 ± 0,7	30,0 ± 3,3	61,9 ± 8,6
p-value vs untreated *rd2*	*0,051*	** *0,001*	** *0,005*
*rd10* untreated	TUNEL	PKM2	Glut1
Sample 1	11,1	9,5	29,1
Sample 2	9,5	10,6	49,9
Sample 3	11,0	14,8	42,1
Sample 4	30,4	52,7	32,3
Sample 5	20,7	54,7	31,7
Mean ± SD	16,5 ± 8,9	28,4 ± 23,1	37,0 ± 8,7
*rd10* + TEPP-46	TUNEL	PKM2	Glut1
Sample 1		48,4	18,1
Sample 2	5,2	60,3	19,4
Sample 3	7,4	57,6	25,1
Sample 4	4,4		43,0
Sample 5	7,0		43,3
Mean ± SD	6,0 ± 1,4	55,5 ± 6,2	29,8 ± 12,5
p-value vs untreated *rd10*	* *0,028*	* *0,029*	*0,830*

**Figure 3.**
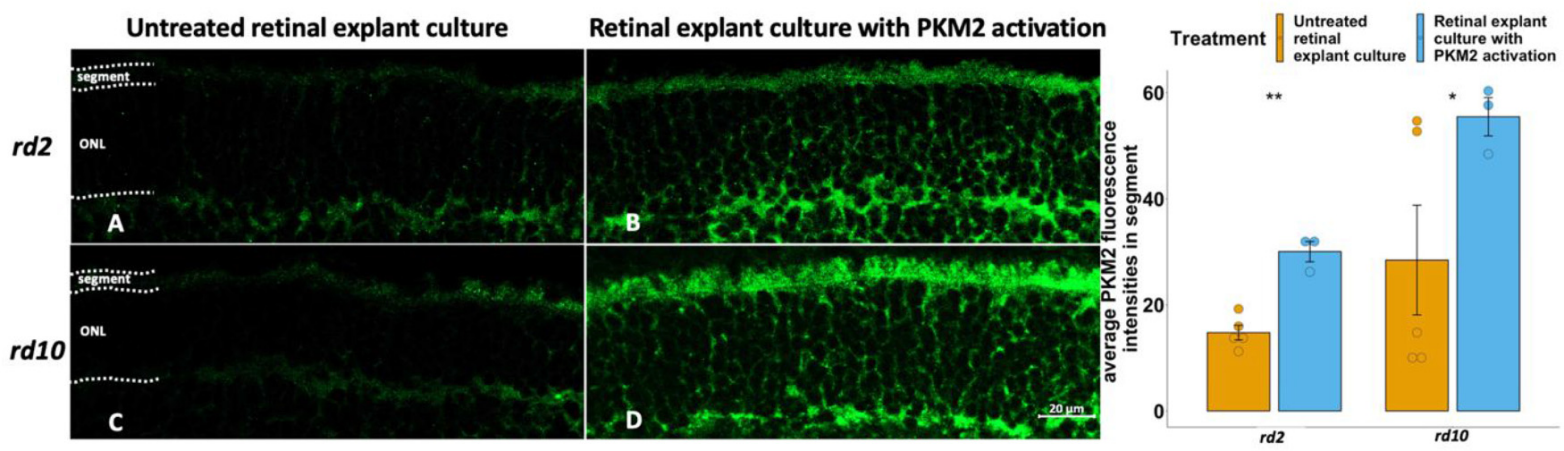
Activation of PKM2 in retinal explants showed higher PKM2 expression in *rd2* and *rd10*. PKM2 fluorescence staining is in green, and DAPI (blue) was used as a nuclear counterstain. A: *rd2* untreated control; B: *rd2* treated with TEPP-46 (PKM2 activator); C: *rd10* untreated control; D: *rd10* treated with TEPP-46; E: Bar chart summarizing the PKM2 fluorescence intensities at segments of the different experimental groups, respectively. The t-test was used as described in Materials and methods section, n = 3–5 biological replicates. *p < 0.05, **p < 0.01.

To further analyze whether the neuroprotective effects in degenerating photoreceptors observed are related to PKM2, we also studied the PKM2 expression in TEPP-46 treated, or untreated, explants. Since we had observed that PKM2 is mainly expressed in the segments, we, therefore, focused on the PKM2 expression in this area. We noticed a higher PKM2 expression in the segment from these two RP models than in their untreated counterparts (Figure 3, Table 2), which in both cases amounted to an approximate doubling of the values. We furthermore saw that two of five values for the untreated *rd10* were much higher (3–5 times) than the rest in the group (Table 2). If the PKM2 level in *rd10* relates to the state, or length, of degeneration, as suggested by the results in Figure 1, then these specimens might for some reason display an exaggerated degeneration response. This would go well with that the same specimens also had 2–3 times more TUNEL-positive cells than the others (Table 2). When we tested to omit these samples from the calculations, the effect on PKM2 expression by TEPP-46 on the *rd10* explants rose to more than 4 times of that of the untreated (not shown).

**Figure 4.**
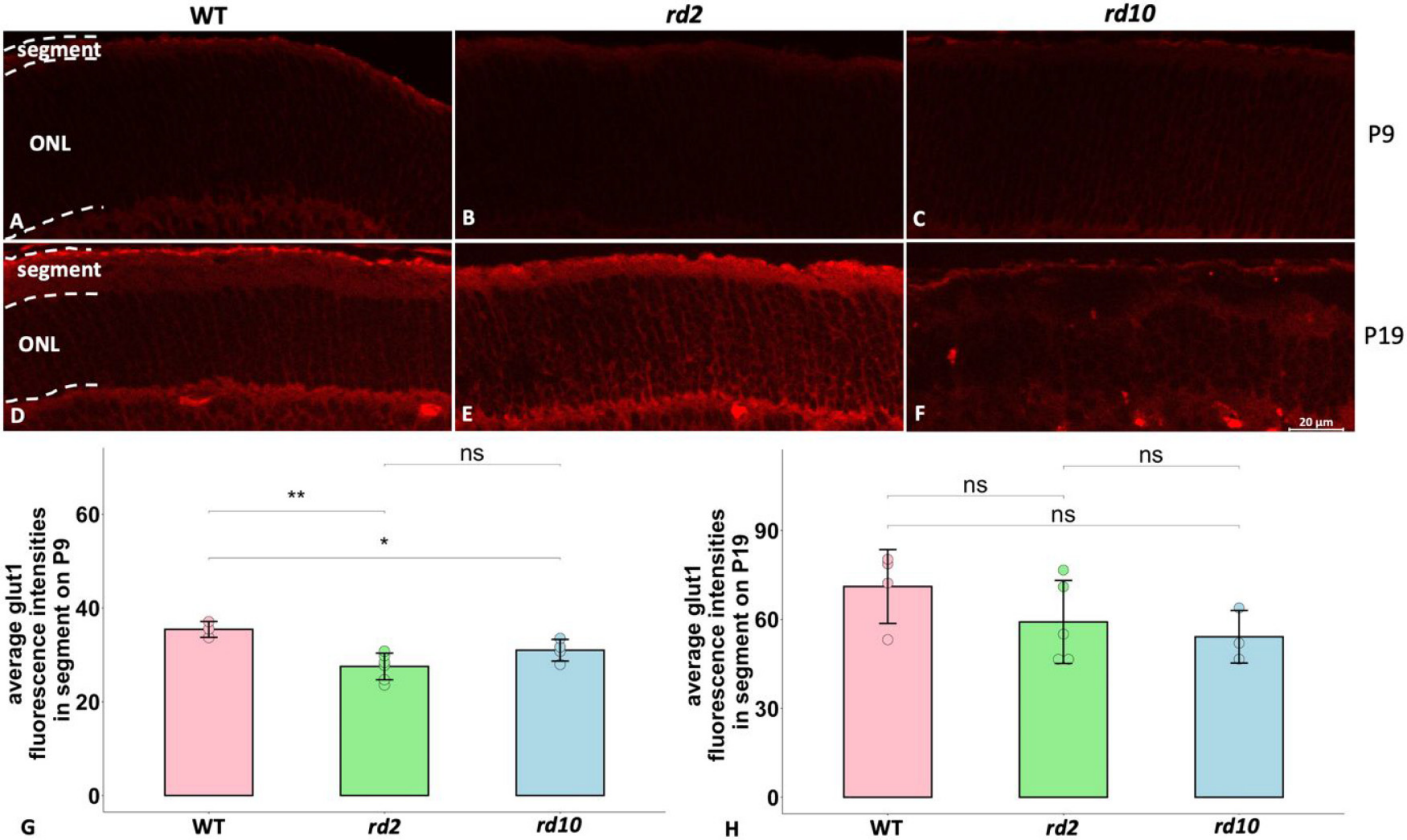
Comparisons of Glut1 expression among WT, *rd2,* and *rd10* on P9 and P19. Glut1 immunofluorescence is shown in red. A-C: PKM2 expression in ONL and segments from WT, *rd2*, and *rd10* at P9; D-F: PKM2 expression in ONL and segments from WT, *rd2*, and *rd10* at P19; G-H: Bar chart summarizing the PKM2 fluorescence intensities among the three different strains on P9 and P19, respectively. The ANOVA with Dunnett's post hoc analysis was used, N = 3–6 biological replicates. *p < 0.05, **p < 0.01. ONL = outer nuclear layer.

To approach how also the PKM2 downstream activities were affected in the degenerating photoreceptors, we next studied how a known PKM2 target, namely glucose transporter-1 (Glut1; Luo& Semenza, 2012), which is essential for rod photoreceptors (Daniele et al., 2022), was expressed in the RP models. As we found that Glut1 was mainly seen within the segments of photoreceptors, we then compared the Glut1 expression within this area between the models and the WT. Here we noticed a lower Glut1 expression in *rd2* and *rd10* than in their WT controls at P9 (Figure 4A–C, G). At P19 we could not see any significant difference in Glut1 expression among these strains (Figure 4D–F, H), although both the *rd2* and the *rd10* showed a numerically reduced level of Glut 1 signal.

**Figure 5.**
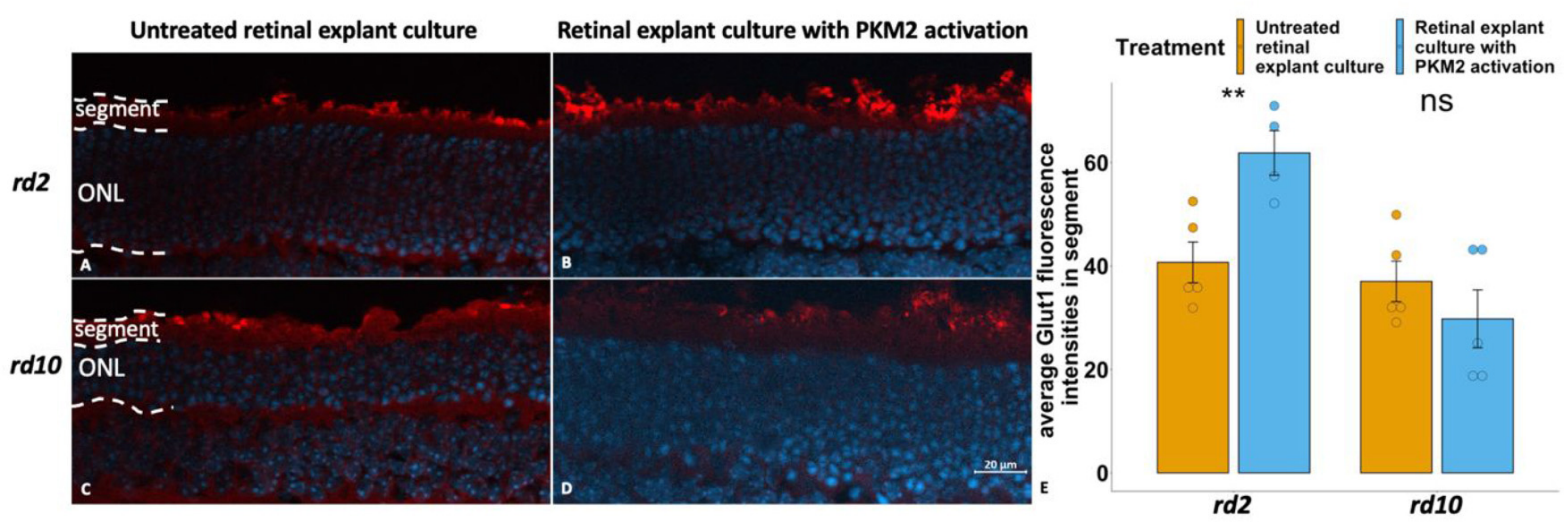
Activation of PKM2 showed an increase of Glut1 expression in segments in *rd2* explants, while the same treatment did not change this parameter in *rd10* explants. Glut1 immunostaining is shown in red, and DAPI (blue) was used as nuclear counterstain. A: *rd2* untreated control; B: *rd2* treated with TEPP-46 (PKM2 activator); C: *rd10* untreated control; D: *rd10* treated with TEPP-46; E: Bar chart summarizing the Glut1 fluorescence in segments of the different experimental groups, respectively. The t-test was used as described in Materials and methods section, n = 4–5 biological replicates. **p < 0.01.

We then investigated if Glut1 expression in the RP models was affected by PKM2 activation with the aid of the organotypic retinal explant cultures. Here we observed that PKM2 activation led to an approximately 50% higher Glut1 expression in the segments in *rd2* than in their untreated peers (Figure 5A–B, E; Table 2). For *rd10*, no significant difference of Glut1 expression was noticed after PKM2 activation (Figure 5C–D, E; Table 2), in spite of a numerical decrease of this parameter in the *rd10* samples.

## Discussion

We hypothesized that PKM2 may work as one of the cGMP-PKG downstream targets in RP and here approached this with the aid of two RP models, *rd2* and *rd10*, that both have an abnormally high level of photoreceptor cGMP (Arango-Gonzalez et al., 2014; Paquet-Durand et al., 2009; Yang et al., 2020). Their retinas were used in combination with an organotypic retinal explant system and the results suggest that PKM2 is connected to photoreceptor degeneration in both models, although with some differences between them in the characteristics of this.

The ONL and segment expression of PKM2 was lower in both of the models than in their WT counterparts at the early age point (P9), whereas at the later age of P19 we noticed a similar lower PKM2 expression in *rd2*, but a clearly higher such in *rd10*. This was to a considerable extent compatible with our hypothesis, which included that the high cGMP in these models via activation of PKG would downregulate the PKM2 levels, just as pharmacological PKG activation downregulated PKM2 gene expression in WT explants (Zhou et al., 2021). Since we also know that similar PKG activation in WT causes retinal degeneration (Paquet-Durand et al., 2009) a reduced PKM2 in the models would be consistent with a critical role for PKM2 for photoreceptor health. For the divergent P19 *rd10*, where PKM2 instead was increased, one must remember that the internally driven cGMP increase in the *rd10* retina, leading to its degeneration, may not necessarily be fully replicated by the more specific and pharmacologically induced PKG activation in WT, which caused a downregulation of PKM2 gene expression (Zhou et al., 2021). The ONL thickness of *rd10* on P19 showed a clear reduction, signifying an ongoing and effective degeneration mechanism with a considerable loss of photoreceptors. At this point the increased PKM2 expression may thus represent a compensating (but failing) effort to counteract the degeneration of the remaining cells, and which somehow bypassed the cGMP-regulation. In fact, PKM2 appears to regulate PDE6β expression (Rajala et al., 2018) and the strong PKM2 upregulation could thus represent an effort to have the missing gene product back. If so, the continuous lower PKM2 expression in *rd2* at P19, an age which shows only a slight decrease in ONL thickness compared to P9, could mean that a similar compensating effects had not started yet, or that the *rd2* pathology as such did not elicit such a response. The latter seems reasonable knowing that the *rd2* degeneration comes from a mutation in gene different from PDE6 (Travis et al., 1991). In either case, this could contribute to why the pharmacological PKM2 activation did not alleviate photoreceptor degeneration in this strain. It should be noted, though, that there are no data on whether the increased expression also leads to increased PKM2 activity, meaning that other events, cGMP related or not, could prevent its full activation.

The lower count of degenerating photoreceptors in *rd10* explants after PKM2 activation indicated a neuroprotective potential of PKM2 during RP progression in this model. For the *rd2* strain, no such change was observed, although the related p value of 0.051 was very close to the statistical threshold. The *rd2* retina has a peak of cell death at around P18, but cell death is seen also several days after that (Arango-Gonzalez et al., 2014). Given that our explant culturing protocol starts at P9 and ends at P19, we might thus have missed any protective effect that may require longer treatment to be detected. Along the same lines, the higher percentage of dying photoreceptors in the *rd10* retina at around P18 (Arango-Gonzalez et al., 2014) could explain why the effect came out clear in that model, together with that the increased PKM2 (see above) could have provided more target for TEPP-46 to activate in *rd10*.

Our observations also included that both *rd2* and *rd10* explants had higher mean PKM2 expression values when treated with TEPP-46, which also alleviated the degeneration in *rd10*. This could relate to that the PKM2 activation provided the photoreceptors with more possibilites to survive, and for *rd10*, where PKM2 increased with time, this manifested in an even higher expression of PKM2. As mentioned in the Results, two of the untreated explants had PKM2 expression levels in the segments that were similar to the peers with PKM2 activation, while the remaining untreated had lower expression. The high PKM2 specimens also had TUNEL values in a clearly higher range, indicating that the PKM2 response could be linked to the degeneration intensity, although we do not know whether this also included an increased PKM2 activity.

Rajala et al. (2018) observed an increased number of degenerating cells within the ONL in retinas from their mouse model with genetically deleted PKM2 expression, which—just as our data here—indicates its importance for photoreceptor viability. Interestingly, they also observed that PKM2 deletion lowered the expression of glucose transporter-1 (Glut1), which has the important function to deliver glucose to photoreceptors (Pragallapati & Manyam, 2019). Our present findings go very well with these discoveries, as we observed Glut1 as well as PKM2 to be lower expressed in both of the RP models compared to WT at an early age. In turn this points to that the PKM2 functions could include regulation of Glut1, in accord with the study of Rajala et al. (2018).

At the later timepoint, P19, the correlation between PKM2 and Glut1 expression was no longer evident in the *rd10,* which at this age had a clearly increased PKM2 level, while its Glut1 level was not correspondingly elevated. In fact, for both *rd2* and *rd10* the average Glut1 levels were, if anything, lower than in WT. A discrepancy between the models was also seen when the effect of PKM2 activation on Glut1 expression was studied, since in this case the activation clearly increased GLUT1 in the *rd2* segments, but not significantly so in *rd10*.

We are thus faced with a situation where both models show distinct, but not identical, connections between the degeneration and PKM2 expression, which compared to WT was reduced in both at P9, but clearly increased in *rd10* at P19. Both models also present connections between Glut1 and the degeneration and/or PKM2, since Glut1 was lower than in WT at P9 in both *rd2* and *rd10*, but here the PKM2 activator gave an increase of Glut1 only in *rd2*.

It is not possible at this stage to fully explain these dissimilarities, and judge what they mean for the prospect to protect the photoreceptors via PKM2 activation. As mentioned above, the lack of protection in *rd2*, at least in the paradigm used here, could be a result of the degeneration characteristics of this model. However, as an alternative explanation, the observed increase in PKM2 in *rd10*, which must have happened between P9 and P19, could represent a time-dependent, inherent protective response. Such a response was in our set-up not detected in *rd2*, meaning that the high level of the *rd10* PKM2 enzyme then may have provided a much better starting point for the work of the PKM2 activator in the *rd10* explants than in those from the *rd2* model. Still the PKM2 activator was able to increase Glut1 in *rd2* explants, but Glut1 is not the only protein that PKM2 interacts with (Luo & Semenza, 2012). This means that other proteins, with a potential bearing on photoreceptor survival, may have failed to react to the PKM2 activation, or simply just not reacted enough yet, and thus being unable to provide protection.

The role of PKM2 may be to stabilize photoreceptors via the maintenance of energy supply, a situation that appears vital as photoreceptors are regarded as one of the most metabolically active cells within the body (Country, 2017). In the retinal ecosystem, the photoreceptors, similar to cancer cells, mainly perform so-called aerobic glycolysis to meet the high energy requirement (Rajala, 2020), and PKM2 acts as a key regulator of glycolysis (Gupta & Bamezai, 2010). Though the ATP generation rate is lower in aerobic glycolysis than in the tricarboxylic acid cycle (TCA), the aerobic glycolysis product pyruvate can be brought to the retinal pigment epithelium (RPE) and converted to lactate and enter the TCA for a higher ATP production (Rajala, 2020). In fact, the general outcome of our previous transcriptome analysis (Zhou et al., 2021) was that an overactive cGMP system could be an important negative regulator of retinal metabolic events. Since increased cGMP levels can be linked to many types of RP (Power et al., 2020), it is thus possible that disturbed actions of PKM2, or other enzymes related to energy metabolism, lie at the heart of the RP pathology.

PKM2 may have yet other roles, since the glycolysis pathway in photoreceptors is not eliminated in case of PKM2 deletion, although further details are unclear (Rajala et al., 2018). Apart from its role in providing energy, aerobic glycolysis has distinct advantages in photoreceptors, since it can benefit photoreceptors via carbon intermediates generation with varied functions, some of which are essential for daily outer segment renewal (Léveillard, 2015). Also, PKM could have more metabolism reprogramming effects, for instance to redirect glucose metabolism to other signaling, which is essential for amino acid production (Zhang et al., 2019). The questions of how these non-glycolysis functions of PKM2 contribute to the photoreceptors’ wellbeing yet require further studies.

In conclusion, while many questions with respect to PKM2 and photoreceptor degeneration remain to be answered, like if its retinal substrates are the same as in other tissues, and if this changes in disease, as well as if PKM2 inhibition of WT explants will mimic the degenerative outcome of genetic deletion of the enzyme (Rajala et al., 2018), the results from our study shed light on a potential, novel molecular target that may exert a direct effect on RP.

## References

[bibr1-17590914231151534] Arango-Gonzalez B. Trifunović D. Sahaboglu A., et al. (2014). Identification of a common non-apoptotic cell death mechanism in hereditary retinal degeneration. PLoS One, 9, 112142. 10.1371/journal.pone.0112142 PMC4230983 25392995

[bibr2-17590914231151534] Chang B. Hawes N. Hurd R., et al. (2002). Retinal degeneration mutants in the mouse. Vision Research, 42, 517–525. 10.1016/S0042-6989(01)00146-8 11853768

[bibr3-17590914231151534] Country M. (2017). Retinal metabolism: A comparative look at energetics in the retina. Brain Research, 1672, 50–57. 10.1016/j.brainres.2017.07.025 28760441

[bibr4-17590914231151534] Daiger S. Sullivan L. Bowne S. (2013). Genes and mutations causing retinitis pigmentosa. Clinical Genetics, 84, 132–141. 10.1111/cge.12203 23701314 PMC3856531

[bibr5-17590914231151534] Daniele L. L. Han J. Y. S. Samuels I. S., et al. (2022). Glucose uptake by GLUT1 in photoreceptors is essential for outer segment renewal and rod photoreceptor survival. FASEB Journal, 36(8), e22428. 10.1096/fj.202200369R 35766190 PMC9438481

[bibr6-17590914231151534] Gupta V. Bamezai R. N. K. (2010). Human pyruvate kinase M2: A multifunctional protein. Protein Science, 19(11), 2031–2044. 10.1002/pro.505 20857498 PMC3005776

[bibr7-17590914231151534] Haim M. (2002). The epidemiology of retinitis pigmentosa in Denmark. Acta Ophthalmologica Scandinavica, 80, 1–34. 10.1046/j.1395-3907.2002.00001.x 11921605

[bibr8-17590914231151534] Jiang J. Boxer M. Vander Heiden M., et al. (2010). Evaluation of thieno[3,2-b]pyrrole[3,2-d]pyridazinones as activators of the tumor cell specific M2 isoform of pyruvate kinase. Bioorganic Med Chem Lett, 20, 3387–3393. 10.1016/j.bmcl.2010.04.015 PMC2874658 20451379

[bibr9-17590914231151534] Luo W. Semenza G. (2012). Emerging roles of PKM2 in cell metabolism and cancer progression. Trends in Endocrinology & Metabolism, 23(11), 560–566. 10.1016/j.tem.2012.06.010 22824010 PMC3466350

[bibr10-17590914231151534] Léveillard T. (2015). Cancer metabolism of cone photoreceptors. Oncotarget, 6(32), 32285–32286. 10.18632/oncotarget.5963 26450906 PMC4741680

[bibr11-17590914231151534] Paquet-Durand F. Hauck S. van Veen T., et al. (2009). PKG Activity causes photoreceptor cell death in two retinitis pigmentosa models. Journal of Neurochemistry, 108, 796–810. 10.1111/j.1471-4159.2008.05822.x 19187097

[bibr12-17590914231151534] Power M. Das S. Schütze K., et al. (2020). Cellular mechanisms of hereditary photoreceptor degeneration - focus on cGMP. Progress in Retinal and Eye Research, 74, 100772. 10.1016/j.preteyeres.2019.07.005 31374251

[bibr13-17590914231151534] Pragallapati S. Manyam R. (2019). Glucose transporter 1 in health and disease. Journal of Oral and Maxillofacial Pathology, 23, 443. 10.4103/jomfp.JOMFP_22_18 31942129 PMC6948067

[bibr14-17590914231151534] Rajala A. Wang Y. Brush R., et al. (2018). Pyruvate kinase M2 regulates photoreceptor structure, function, and viability. Cell Death & Disease, 9, 240. 10.1038/s41419-018-0296-4 29445082 PMC5833680

[bibr15-17590914231151534] Rajala R. V. (2020). Aerobic glycolysis in the retina: Functional roles of pyruvate kinase isoforms. Frontiers in Cell and Developmental Biology, 8. 10.3389/fcell.2020.00266 PMC7203425 32426353

[bibr100-17590914231151534] Sanyal, S., & Bal, A. K. (1973). Comparative light and electron microscopic study of retinal histogenesis in normal and RD Mutant Mice. *Zeitschrift für Anatomie und Entwicklungsgeschichte, 142*(2), 219–238. 10.1007/bf00519723 4781863

[bibr16-17590914231151534] Sanyal S. De Ruiter A. Hawkins R. K. (1980). Development and degeneration of retina in rds mutant mice: Light microscopy. The Journal of Comparative Neurology, 194(1), 193–207. 10.1002/cne.901940110 7440795

[bibr17-17590914231151534] Travis G. H. Sutcliffe J. G. Bok D. (1991). The **retinal degeneration** slow (**rds**) **gene** product is a photoreceptor disc membrane-associated glycoprotein. Neuron, 6, 61–70. 10.1016/0896-6273(91)90122-G 1986774

[bibr18-17590914231151534] Vighi E. Trifunović D. Veiga-Crespo P., et al. (2018). Combination of cGMP analogue and drug delivery system provides functional protection in hereditary retinal degeneration. Proceedings of the National Academy of Sciences of the USA, 115, 2997–3006. 10.1073/pnas.1718792115 PMC5879685 29531030

[bibr19-17590914231151534] Yang P. Lockard R. Titus H. Hiblar J. Weller K. Wafai D. Weleber R. Duvoisin R. Morgans C. Pennesi M. (2020). Suppression of cGMP-dependent photoreceptor cytotoxicity with mycophenolate is neuroprotective in murine models of retinitis pigmentosa. Investigative Opthalmology & Visual Science, 61(10), 25. 10.1167/iovs.61.10.25 PMC7441375 32785677

[bibr20-17590914231151534] Zhang Z., et al. (2019). PKM2, function and expression and regulation. Cell & Bioscience, 9(1). 10.1186/s13578-019-0317-8 PMC6595688 31391918

[bibr21-17590914231151534] Zhou J. Rasmussen M. Ekström P. (2021). cGMP-PKG dependent transcriptome in normal and degenerating retinas: Novel insights into the retinitis pigmentosa pathology. Experimental Eye Research, 212, 108752. 10.1016/j.exer.2021.108752 34478738

